# Development and testing of a questionnaire on the expectations and experiences with wearing face masks in inpatient and day hospitals

**DOI:** 10.1371/journal.pone.0304140

**Published:** 2024-05-31

**Authors:** Charlotte Kuczyk, Kathrin Münch, Mariel Nöhre, Michael Stephan, Martina de Zwaan

**Affiliations:** Department of Psychosomatic Medicine and Psychotherapy, Hannover Medical School, Hannover, Germany; Medical University of Vienna, AUSTRIA

## Abstract

**Background:**

The Covid-19 pandemic made wearing of face masks mandatory in the psychotherapeutic context. Against this background, the present study aimed to compare the expectations of patients undergoing day-hospital or inpatient treatment regarding wearing a mask in psychotherapy before the start of therapy with the final experience after the end of therapy. The study also investigated the extent to which expectations and experiences were influenced by other factors such as socio-demographic characteristics, patients’ general attitudes towards wearing a mask, duration of treatment, or mental health diagnoses.

**Methods:**

Patients’ expectations and experiences were recorded using two versions of a self-developed questionnaire: the pre-version, which was administered before the start of therapy and recorded expectations, and the post-version, which was administered after the end of therapy and recorded the final experiences. An exploratory factor analysis was conducted for the questionnaire’s pre- and post-version. T-tests for paired samples were calculated to compare the patients’ expectations regarding the extracted factors with the final experiences. Bivariate correlations were calculated to explore the association of other potential factors with expectations and experiences.

**Results:**

The exploratory factor analysis revealed a three-factor structure: *communication barriers*, *self-confidence*, and *infection protection*. The communication barriers expected by the patients before the start of the therapy turned out to be significantly higher than ultimately experienced after the therapy. Higher age correlated significantly negatively with expectations and experiences, with less self-confidence expected and experienced in therapy with a mask by older patients. There was a significant positive correlation between the expectations and the duration of treatment. Patients’ general attitudes correlated significantly with their expectations and experiences.

**Conclusion:**

Based on the results, wearing a mask does not appear to negatively impact the success of psychotherapy from the patient’s perspective. However, patient-specific characteristics also appear to play a role in this context.

## Introduction

The COVID-19 pandemic led to massive changes and restrictions in everyday life and brought significant uncertainties regarding the delivery of mental health services. Particularly in the field of mental health care, professionals faced a major challenge, as wearing a face mask was associated with significant limitations in verbal and non-verbal communication on both the patient and therapist side [1–3]. In particular, non-verbal communication restricted by the mask [4] appeared to be particularly problematic in the psychotherapeutic context, as non-verbal behavior (facial expressions, gestures, and posture) has been shown in previous research to be essential for the development of an affective bond between patient and therapist and thus, among other factors, for the development of a working alliance [5,6]. For example, Hall et al. [7], in their review of research on non-verbal behavior in patient-therapist interaction, emphasized the importance of active facial expressions for the development of an affective bond, for example, in the form of a moderate smile on the part of the therapist.

Furthermore, emotion work forms an essential part of almost all forms of psychotherapy [8,9], whereby the face has proven to be an essential informative visual stimulus in the human perception of emotions [10]. The high social visibility, accessibility, and expressive power of the face make it a crucial medium for exchanging emotional information [11,12]. The latter raises the crucial question, particularly for the psychotherapeutic context, of the extent to which emotion recognition based on the face could be impaired by facial masking, for example, in the form of a face mask, thus limiting the success of psychotherapy.

### Influence of facial masks on facial expressions as an expression of emotionality

However, in nonverbal communication, wearing face masks significantly restricts facial expressions, which seems particularly problematic since facial expressions are significant for successfully decoding emotions [13]. All emotion expressions are generally mapped to the face via a specific pattern of activity [14], with increased muscle activity in the zygomaticus major muscle (associated with smiling) observed for facial expressions with positive valence and increased muscle activity in the corrugator supercilii (associated with frowning) for facial expressions with negative valence [15]. Consistent with the latter, previous research showed that when facial expressions were experimentally blocked, the recognition of different emotional expressions was impeded or prevented, depending on the particular area manipulated. For example, Borgomaneri et al. [16] showed in their study that manipulating the facial expressions of the lower visual field prevented the recognition of happy facial expressions, while the recognition of other emotions was unaffected by the latter.

The latter is consistent with the study of Gosselin et al. [17], in which facial area manipulation was used to show that the expression of happiness manifested exclusively in the lower facial area. In contrast, expressions of fear and sadness involved more upper than lower facial areas [18–20], whereas expressions of surprise involved both upper and lower facial areas [21]. In the study by Maiorana et al. [22], sadness and anger proved to be the most frequently misinterpreted emotions when only the eye region was visible, whereby the authors were able to show in a reaction time analysis that emotion recognition was significantly slower overall in the presence of a face mask than when the entire facial area was visible. Tsantani et al. [23], in turn, showed in their studies that the partial covering of the face using a surgical face mask made the perception of emotional expressions such as joy, sadness, anger, fear, disgust, and surprise equally difficult.

The studies described above have in common that the investigation of emotion perception was carried out under experimental conditions using an isolated representation of the face. In this context, however, it must be considered that in social interaction situations, faces are rarely viewed in isolation but are usually accompanied by body language, vocal cues, hand gestures, and posture. In other studies that take a holistic view of the body in the context of emotion recognition, there are indications that when the expression of emotionality via the face is limited, other cues with similar ability to recognize emotions are increasingly focused on. In this context, Ross and George [24], for example, showed in their study that the recognition of emotions in the absence of facial information is also reliably possible based on body language alone.

It should also be noted that emotions are communicated through non-verbal prosodic cues, including intonation, pitch, and emphasis of the speaker’s vocal production [25]. For example, different emotions have different prosodic profiles, which listeners can use to determine the speaker’s emotional state. Consistent with the latter, Juslin et al. [26] showed that participants could recognize and name different emotions with a high degree of accuracy using only audio clips containing natural conversations.

Consequently, there is increasing evidence in the literature that losing information in the lower part of the face when wearing a face mask could lead to a significant loss of information crucial for distinguishing between basic emotions. However, the extent to which emotion recognition of different emotions is equally influenced by wearing a face mask or rather the recognition of specific emotions, the expression of which is possibly more likely to be reflected in the lower half of the face, is not clear from the literature to date. Furthermore, as described above, there are also indications in the literature to date that the influence of masks on emotion recognition may not be as pronounced as previously assumed as long as other cues, such as the overall body image or vocal stimuli, are available that can be used by the observer to recognize emotions.

### Differences depending on individual characteristics

However, there is also evidence in the literature that the extent of the influence of wearing a mask on the psychotherapeutic context may also depend on other factors, such as socio-demographic characteristics, general attitudes toward wearing a face mask, or the patient’s diagnosis. Concerning socio-demographic characteristics, gender plays a role, as females often seem better at decoding emotions, especially based on the eye region [27,28]. Age also plays an important role, as older adults appear less efficient at recognizing facial expressions than younger adults [29]. In this context, for example, older adults have been shown to focus mainly on the lower part of the face during emotion recognition. In contrast, younger adults are more exploratory and repeatedly switch between the lower and upper half of the face [30].

Furthermore, there is evidence that individual differences regarding general attitudes towards wearing mouth and nose protection might affect emotion recognition in masked faces. In this context, Biermann et al. [31], for example, managed to show in their study that a more substantial negative bias in judgments of happiness and trustworthiness based on masked faces was evident among those who generally attributed a lower protective function to the masks, experienced the COVID-19 pandemic as less risky, and perceived wearing a face mask as a substantial burden. Moreover, in their study, when assessing trustworthiness, negative bias was found to be increased among those who tended to be less compliant with the mandatory requirement of wearing a face mask.

Furthermore, with particular relevance to the psychotherapeutic context, there are also indications in the literature to date that the ability to recognize emotions appears to be limited, especially in the case of certain mental disorders. In this context, for example, Unoka et al. [32] were able to show in their study results that patients with BPD were less able to distinguish between different negative emotions than control participants. Furthermore, there is also evidence in the literature to date that BPD patients tend to perceive neutral stimuli (primarily neutral faces) as more negative than healthy controls [33]. Emotion recognition was also found to be significantly more limited and less precise in a sample of patients with existing moderate depression compared to a control group [34]. The latter, in particular, once again illustrates the need to investigate the effects of wearing a face mask on the psychotherapeutic context from the patient’s perspective using a sample characterized by various mental disorders.

### Objective of the study

- The first objective of this study was to assess expectations and experiences from a patient’s perspective regarding wearing face masks during psychotherapy. To date, only a few studies have examined the impact of wearing a face mask on psychotherapeutic work. However, to our knowledge, no study is yet available that assesses patients’ specific expectations about wearing a face mask during psychotherapy before therapy begins and compares them with their immediate experiences after therapy ends.
- The second aim of this study was to investigate the extent to which other factors (patients’ sociodemographic characteristics or general attitudes, thoughts, and feelings about wearing a face mask in public or mental health diagnoses influence patients’ expectations and experiences.

It is essential to know how patients perceive face masks during clinical consultations, whether they can benefit equally from the therapy even with face masks, or to what extent the latter is subject to certain influencing factors. These questions appear to be of great relevance even after the peak phase of the pandemic has subsided, on the one hand, in order to be prepared for future pandemics and, on the other hand, because the use of face masks is still an integral part of clinical activities for both clinicians and patients in many areas.

## Materials and methods

### Participants

The study was conducted at the Hannover Medical School, Department of Psychosomatic Medicine and Psychotherapy. The study was approved by the ethics committee of the Hannover Medical School (ref. no. 9666_BO_K_2021). Data collection took place during the period from March 15, 2021, to November 25, 2021. All patients undergoing day hospital or inpatient treatment during this period were informed about the study and included if they consented. A sample of patients undergoing inpatient or day-care treatment was used to conduct the study, as a strictly controlled standard for wearing mouth and nose protection was in place for patients and practitioners during the pandemic, particularly in hospital settings. All patients undergoing inpatient or day-care treatment received a combination of individual and group psychotherapeutic interventions at the Department of Psychosomatic Medicine and Psychotherapy to treat their respective disorders. All participants provided written informed consent. The only participation requirement was the presence of sufficient German language skills; otherwise, no further exclusion criteria were defined. The patients received no compensation for their participation.

Patient characteristics, such as gender, age, length of hospital stay, and mental health diagnoses at discharge, were taken from the patient record. A sample of N = 142 [mean age = 40.0 years (SD = 13.5), 102 (71.8%) women, 40 (28.25%) men [(78 (54.9%) inpatients and 64 (45.1%) day patients)] was included in the study. Length of hospital stay was, on average, 54.3 (SD = 13.1) days. In terms of ICD-10 diagnoses (multiple diagnoses allowed), depression (ICD-10: F31.3, F32, F33, F34.1) was diagnosed in 133 patients (93.7% of the whole sample); 50 (35.2%) patients were diagnosed with one or more anxiety disorder (F40, F41) or obsessive-compulsive disorder (F42), 69 (48.6%) with one or more somatoform disorder (F44, F45), 43 (30.3%) with an eating disorder (F50), 33 (23.2%) with PTSD, and 35 (24.60%) patients had one or more personality disorder (see Table 1). Most participants received more than one Axis I diagnosis (99.3%), 75.4% had three or more diagnoses, and 15.5% had five or more diagnoses (For an overview of the sample characteristics regarding the diagnosis according to ICD-10, see Table 1).

**Table 1:** Sample characteristics.

Diagnosis according to ICD-10^1^	N	N (%)
**Depression**		
F31.3	1	0.7
F32	16	11.3
F33	116	81.7
F34.1	5	3.5
**Anxiety disorder**		
F40, F40.01	14	9.9
F40.1	11	7.7
F40.2	2	1.4
F41	14	9.9
F41.1	4	2.8
**Obsessive-compulsive disorder**		
F42.0, F42.1, F42.2	9	6.3
**PTSD**		
F43.1	33	23.2
**Somatoform Disorder**		
F44	7	4.9
F45	64	45.1
**Eating disorder**		
F50.0	43	30.3
**Personality Disorder**		
F60	26	18.3
F61	9	6.3
**Other**		
F54	27	19
F9	10	7.0

Notes.

^1^ Not mutually exclusive.

### Item generation and study procedure

In developing the questionnaire, the therapist team addressed possible problems and limitations associated with wearing oral-nasal protection in psychotherapeutic work. Subsequently, items were created from the patient’s perspective that related either to possible limitations of wearing a mask on the part of the patient or to possible limitations of wearing a mask on the part of the therapist. The comprehensibility and relevance of the items were tested on a small group of patients (N = 10) in the sense of a pretest (cognitive debriefing).

Based on this, two versions of a questionnaire (pre- and post-version) were created, each with 27 items with identical content (The questions are listed in S1 Table; the questionnaires are available on request). The pre-version of the questionnaire was given to patients before the start of treatment to ask about their expectations regarding wearing a mask during psychotherapy sessions. The post version of the questionnaire was given to patients at the end of their day hospital or inpatient treatment to record their ultimate experience. To test the validity of the questionnaires, patients were additionally asked at the pre-measurement time point regarding their general attitudes, thoughts, and feelings about wearing a mouth and nose guard in public. Respondents indicated their level of agreement using a 5-point Likert scale with 1 = strongly disagree, 2 = somewhat disagree, 3 = partly agree, 4 = somewhat agree, and 5 = strongly agree. In the missing values analysis, 126 (88.73%) of 142 patients had complete data at the pre-measurement point. At the pre-measurement time, 16 participants (11.27%) showed missing data for one to a maximum of two items. At the post-measurement time, 128 (90.14%) patients had complete data. Fourteen patients (9.86%) had incomplete data, with 12 patients showing missing data on one to a maximum of two items. Two patients had missing values on 11 items at the post-measurement time.

### Statistical analysis

First, a general data inspection was performed. The normal distribution of individual items was checked visually using Q-Q plots, skewness, and kurtosis. Items that were not substantially normally distributed (absolute univariate skewness > 2 and absolute univariate kurtosis > 7) [35] were excluded before analyses were conducted. Missing data were replaced by imputation using the expectation-maximization (EM) algorithm, an iterative maximum likelihood procedure [36–38]. Previous research has shown that maximum likelihood estimation is preferable to more common methods such as pairwise deletion, listwise deletion, or mean substitution when dealing with missing data [39–41]. Exploratory factor analyses were conducted using principal axis factorization with Oblique rotation to examine the underlying factor structure of the pre-version (expectations before therapy began) and post-version of the questionnaire (experiences after therapy ended) to identify temporal changes and test the robustness of the factor solution over time. Oblique rotation was chosen based on the assumption of Tabachnick and Fiddell [42], who argue that “Perhaps the best way to decide between orthogonal and oblique rotation is to request oblique rotation with the desired number of factors and look at the correlations among factors… if factor correlations are not driven by the data, the solution remains nearly orthogonal. Look at the factor correlation matrix for correlations around .32 and above. If correlations exceed .32, there is 10% (or more) overlap in variance among factors, enough variance to warrant oblique rotation unless there are compelling reasons for orthogonal rotation.” To determine the number of factors to retain, several criteria were considered, including (1) an eigenvalue greater than one, (2) visual inspection of the scree plot, (4) parallel analysis [43], (3) an on-factor loading of >0.40, (5) a cross-loading <0.40, and (5) theoretical considerations.

After selecting the appropriate number of factors for the pre-and post-versions of the questionnaire, the factors were combined into subscales by summing the responses of the items associated with a factor for each of the pre-and post-versions. In the process of scale refinement, we evaluated the internal consistency of the scales using Cronbach’s α. T-tests for paired samples were calculated to compare the subscale scores of the pre-and post-version to compare expectations about wearing a mask in psychotherapy with the ultimate experience. To test the validity of the defined subscales and to investigate the association with other factors on patients’ expectations and experiences, Pearson correlations were calculated between the subscales of the pre-and post-versions of the questionnaire and the general attitudes, thoughts, and feelings about wearing a face mask in public collected at the pre-time point. In addition, Pearson correlations were calculated between the subscales of the pre-and post-versions of the questionnaire and the age and length of therapy. Point-biserial correlations were calculated between the subscales and gender and mental health diagnoses. All analyses were performed using IBM SPSS 28 software (IBM SPSS Statistics, 2021).

As described in the section on item generation and study procedure, many values were missing at the post-measurement time point in two cases. To check the robustness of the results, sensitivity analyses were performed by calculating all analyses for the post-measurement again without the two cases mentioned.

## Results

### Exploratory factor analyses

Five of the original 27 items were initially excluded due to a highly divergent distribution (i.e., absolute univariate skewness > 2 and absolute univariate kurtosis > 7), so that 22 variables are included in the following analyses for each of the pre-and post-versions of the questionnaire. To test the suitability of the items for the exploratory factor analyses (EFA), the Kaiser-Meyer-Olkin (KMO) test and Bartlett’s test for sphericity were performed. The KMO score was 0.79 when the pre-version items were included and 0.82 when the post-version items were included, indicating that the items were suitable for conducting the exploratory factor analyses [44]. Bartlett’s test for sphericity indicated item correlations for the pre-version (χ^2^(190) = 1281.24, p < .001) and for the post-version (χ^2^(231) = 1630.87, p < .001) that had a magnitude acceptable for EFA. The determinants of the correlation matrix indicated no problems with multicollinearity among items for the pre- and post-versions (determinant >. 001). In the first round of exploratory factor analyses for pre- and post-version, the principal axis factoring and Oblique rotation of each of the 22 items resulted in five factors with eigenvalues greater than 1.

However, the parallel analyses and the scree plots indicated a three-factor solution. On this basis, a three-factor solution was chosen in each case, explaining 44.53% of the total variance in the pre-version and 48.80% in the post-version. The overview of the loadings of the initial three-factor solution can be found for the pre-version in the S2 Table and the post-version in the S3 Table. After applying the item selection criteria (an on-factor loading of >0.40), two items (12,11) were excluded from both the pre-version and the post-version. Because item 27 showed a loading < .04 on Factor 1 at the pre-version, but this item showed a loading of .06 on Factor 1 at the post-version, it was decided to retain this item. However, to keep the subscales consistent across both questionnaire versions for subsequent analyses, this item was not included in the subsequent calculation of subscale scores. In the context of testing the internal consistency of the subscales, the exclusion of item 14 in the post-version on Factor 1 resulted in a significant improvement in Cronbach’s α (α = .912 to α = .915), so we decided to exclude this item in the construction of the scale for both the pre-and post -version, again to maintain consistency and improve the psychometric properties of the scale. The final three-factor solution resolved 48.81% of the total variance for the pre-version (see Table 2) and 53.30% for the post-version (see Table 3).

**Table 2:** Three-factor solution and descriptive statistics for pre-measurement (Items 11, 12 and 14 excluded).

	Factor loadings									
	1	2	3							
Eigenvalue	4.89	3.05	1.33							
Variance explained	25.74%	16.06%	7.01%							
Item				*h* ^2^	M	Mdn	SD	Skewness	Kurtosis	Min/Max
26	**.778**	.056	.020	.60	3.31	3.0	1.15	-.18	-.55	1/5
4	**.770**	.000	.122	.55	3.21	3.0	1.15	-.34	-.56	1/5
1	**.767**	-.059	.116	.54	2.53	2.0	1.21	.35	-.94	1/5
2	**.701**	-.038	.000	.49	2.15	2.0	1.06	.70	-.14	1/5
19	**.687**	-.068	-.070	.51	2.41	2.0	1.22	.40	-.90	1/5
3	**.672**	-.020	-.027	.44	3.30	3.0	1.21	-.21	-.99	1/5
16	**.626**	-.002	-.027	.40	2.89	3.0	1.16	-.04	-.82	1/5
23	**.610**	.008	-.030	.40	1.72	1.0	0.94	1.22	.76	1/5
25	**.586**	-.013	-.041	.36	3.29	3.0	1.18	-.28	-.74	1/5
28	**.456**	-.041	-.095	.25	3.36	3.0	1.18	-.15	-.82	1/5
27	.326	.205	-.245	.26	2.38	2.0	1.22	.56	-.69	1/5
7	-.100	**.802**	-.001	.64	1.88	1.0	1.13	1.10	.16	1/5
8	.068	**.746**	-.026	.57	2.04	2.0	1.13	.96	.16	1/5
9	-.099	**.719**	-.002	.52	1.66	1.0	0.94	1.44	1.57	1/5
6	.076	**.675**	.160	.50	2.12	2.0	1.18	.90	-.13	1/5
22	.044	**.609**	-.057	.38	1.77	1.0	0.99	1.12	.31	1/5
10	-.051	**.597**	.036	.36	2.33	2.0	1.38	.55	-1.00	1/5
24	.046	.108	**.914**	.84	3.42	4.0	1.43	-.50	-1.05	1/5
5	-.034	.013	**.806**	.67	3.62	4.0	1.27	-.66	-.55	1/5
Factor correlations										
1.	-	-	**-**							
2.	.049	-	**-**							
3.	-.322	.078	**-**							

*Notes*. N = 142. Loadings from the rotated solution (Oblique) with Kaiser’s normalization, principal axes factoring. M = mean, Mdn = median, SD = standard deviation, Min/Max = minimum and maximum value of the response scale. Values highlighted in bold indicate factor loadings >.40.

**Table 3:** Three-factor solution and descriptive statistics for the post-measurement (Items 11, 12 und 14 excluded).

	Factor loadings									
	1	2	3							
Eigenvalue	4.89	3.05	1.33							
Variance explained	25.74%	16.06%	7.01%							
Item				*h* ^2^	M	Mdn	SD	Skewness	Kurtosis	Min/Max
26	**.778**	.056	.020	.60	3.31	3.0	1.15	-.18	-.55	1/5
4	**.770**	.000	.122	.55	3.21	3.0	1.15	-.34	-.56	1/5
1	**.767**	-.059	.116	.54	2.53	2.0	1.21	.35	-.94	1/5
2	**.701**	-.038	.000	.49	2.15	2.0	1.06	.70	-.14	1/5
19	**.687**	-.068	-.070	.51	2.41	2.0	1.22	.40	-.90	1/5
3	**.672**	-.020	-.027	.44	3.30	3.0	1.21	-.21	-.99	1/5
16	**.626**	-.002	-.027	.40	2.89	3.0	1.16	-.04	-.82	1/5
23	**.610**	.008	-.030	.40	1.72	1.0	0.94	1.22	.76	1/5
25	**.586**	-.013	-.041	.36	3.29	3.0	1.18	-.28	-.74	1/5
28	**.456**	-.041	-.095	.25	3.36	3.0	1.18	-.15	-.82	1/5
27	.326	.205	-.245	.26	2.38	2.0	1.22	.56	-.69	1/5
7	-.100	**.802**	-.001	.64	1.88	1.0	1.13	1.10	.16	1/5
8	.068	**.746**	-.026	.57	2.04	2.0	1.13	.96	.16	1/5
9	-.099	**.719**	-.002	.52	1.66	1.0	0.94	1.44	1.57	1/5
6	.076	**.675**	.160	.50	2.12	2.0	1.18	.90	-.13	1/5
22	.044	**.609**	-.057	.38	1.77	1.0	0.99	1.12	.31	1/5
10	-.051	**.597**	.036	.36	2.33	2.0	1.38	.55	-1.00	1/5
24	.046	.108	**.914**	.84	3.42	4.0	1.43	-.50	-1.05	1/5
5	-.034	.013	**.806**	.67	3.62	4.0	1.27	-.66	-.55	1/5
Factor correlations										
1.	-	-	**-**							
2.	.049	-	**-**							
3.	-.322	.078	**-**							

*Notes*. N = 142. Loadings from the rotated solution (Oblique) with Kaiser’s normalization, principal axes factoring. M = mean, Mdn = median, SD = standard deviation, Min/Max = minimum and maximum value of the response scale. Values highlighted in bold indicate factor loadings >.40.

Factor 1 was referred to as *communication barriers*, which related to aspects of interaction (experiencing distance, establishing trust, facial expressions) and auditory comprehension. This factor comprised ten items in the pre-version and 11 items in the post-version, with loadings between 0.778 and 0.456 (explained variance 25.74%) in the pre-version (see Table 2) and loadings between 0.791 and 0.596 (explained variance 31.47%) in the post version (see Table 3). The indexed item within the factor was *“Through the therapist’s mask*, *I will not/could not tell how he/she responds to me”* (Item 26) for both the pre and post-versions. Factor 2 was referred to as *self-confidence* induced by the mask. It referred to the influences of the mask on the outer appearance of the wearer (including the possibility of concealing subjective external inadequacies through the mask) and of the observer, as well as to the associated possibilities for opening up in terms of content (e.g., *“Through the mask*, *I could open up more easily and talk about myself”*). This factor comprised six items with loadings between 0.802 and 0.597 (explained variance 16.06%) for the pre-version (see Table 2) and loadings between 0.785 and 0.464 (explained variance 13.87%) for the post-version (see Table 3). The initial item of Factor 2 was *“The mask will make it easier for me to open up and talk about myself”* (Item 7) for the pre-version and *“With a mask*, *I could concentrate better on my issues”* (Item 9) for the post-version. The third factor was labeled protection against infection and consisted of two items related to the mask in terms of a protection factor against infection with coronavirus, with loadings between 0.914 and 0.806 (explained total variance 7.01%) for the pre-version (see Table 2) and loadings between 0.904 and 0.858 (explained total variance 7.96%) for the post-version (see Table 3). The initial item for the pre-version was *“The therapist’s mask will make me feel better protected against infection”* (Item 24); for the post-version, it was *“The mask made me feel safe from infection during therapy*.*”* (Item 5).

Both the extracted factors of the pre- and post-version showed medium correlations among each other. Hence, the choice of oblique rotation, according to Tabachnick and Fiddell [42], retrospectively proved reasonable.

Internal consistency calculations for the pre-version yielded a Cronbach’s α of .866 for Factor 1 and a Cronbach’s α of .840 for Factor 2, indicating good internal consistency between the scales. Because Factor 3 included a scale with only two items, the Spearman-Brown reliability coefficient was calculated to determine internal consistency [45]. Here, a coefficient of .88 was obtained, indicating a high internal consistency of the scale. Regarding the post version, there was also good internal consistency, with a Cronbach’s α of .915 for Factor 1, a Cronbach’s α of .812 for Factor 3, and a Spearman-Brown coefficient of .885 for Factor 3.

### Comparison of expectations with final experience

T-tests for paired samples were calculated to compare the questionnaire’s pre- and post-versions. Post-treatment, experienced communication barriers were perceived to be significantly less problematic than expected at baseline t(141) = 8.567, p < .001, d = .71. No difference was found between expectations and experiences regarding the self-confidence subscale t(141) = .504, p = .615 or the infection prevention subscale t(141) = .579, p = .564.

### Correlations between expectations and experiences and other factors

Pearson correlation coefficients revealed many statistically significant correlations between the newly developed subscales “*communication Barriers*, *self-confidence*, *and infection protection*” of the pre-and post-versions and general attitudes, thoughts, and feelings about wearing a mouth and nose guard in public. The correlations are presented in Table 4. Regarding the scale *communication barriers*, it became apparent that the patients who stated that wearing medical mouth and nose protection is fundamentally important to themselves and to the other person: *1*.*) “I would like to wear a mask in the therapy session*,*” 2*.*) “I want the therapist to wear a mask in the therapy session*,*” 14*.*) “I mainly use FFP-2 masks in everyday life*,*”* expected fewer communication barriers from wearing a face mask in therapy (pre-version) (1. r = -.338, p < .001; 2. r = -.344, p < .001; 14. r = -.261, p = .002) and ultimately experienced them (post-version) (1. r = -.208, p = .013; 2. r = -.231, p = .006; 14. r = -.251, p = .003). Concerning the scale *self-confidence*, it was shown that the patients who stated that they basically feel more comfortable wearing a mask in everyday life/public also expected to feel more confident in the therapeutic setting by wearing a face mask (pre-version): *5*.*) “The mask makes me pay less attention to my appearance in everyday life (e*.*g*., *made-up lips*, *shaving)” 8*.*) “Since my face can hardly be seen through the mask*, *I feel more comfortable in public”* (5. r = .614, p < .001; 8. r = .571, p < .001) and ultimately experienced it that way (post-version) (5. r = .524, p < .001; 8. r = .622, p < .001). Concerning the scale *infection protection*, it was shown that the patients who stated that they also felt protected from infection in everyday life/public places by wearing mouth and nose protection, *9*.*) “The mask protects me from infection” 10*.*) “I take the mask requirement in public places very seriously” 14*.*) “I mainly use FFP-2 masks in everyday life*,*”* expected (pre-version) to be able to protect themselves from infection also in the therapeutic setting by wearing a mask themselves and the wearing a mask on the therapeutic side (9. r = .750, p < .001; 10. r = .485, p < .001, 14. r = .316, p < .001) and ultimately rated this equally after therapy (post-version) (9. r = .507, p < .001; 10. r = 259, p = .002; 14. r = .202, p = .016).

**Table 4:** Table of means (M), standard deviations (SD) of the general opinion on mask wearing collected at the pre-measurement time and correlations between the general opinion and the extracted factors (CB = communication barriers, SC = self-confidence, IP = infection protection) for the pre-measurement and the post-measurement, respectively.

			Pre-version			Post-version		
	M	SD	1. CB	2. SC	3. IP	1. CB	2. SC	3. IP
**1.**	2.56	1.49	**-.338** ^**a**^	**.299** ^**a**^	**.563** ^**a**^	**-.208 (p = .013)**	**.179 (p = .033)**	**.396** ^**a**^
**2.**	2.63	2.48	**-.344** ^**a**^	.128 (p = .130)	**.609** ^**a**^	**-.231 (p = .006)**	.051 (p = .551)	**.421** ^**a**^
**3.**	2.99	1.35	**.332** ^**a**^	.002 (p = .982)	**-.308** ^**a**^	**.286** ^**a**^	.053 (p = .527)	**-.278** ^**a**^
**4.**	3.16	1.31	**.402** ^**a**^	-.102 (p = .227)	**-.360** ^**a**^	**.300** ^**a**^	-.064 (p = .451)	**-.268 (p = .001)**
**5.**	2.24	1.35	.008 (p = .926)	**.614** ^**a**^	.044 (p = .599)	-.024 (p = .779)	**.524** ^**a**^	.054 (p = .527)
**6.**	3.01	1.25	**.442** ^**a**^	.036 (p = .667)	**-.203 (p = .015)**	**.366** ^**a**^	.012 (p = .887)	**-.191 (p = .023)**
**7.**	2.41	1.22	**.514** ^**a**^	.137 (p = .110)	-.128 (p = .134)	**.422** ^**a**^	**.168 (p = .049)**	-.152 (p = .075)
**8.**	2.56	1.34	.065 (p = .448)	**.571** ^**a**^	.061 (p = .471)	.043 (p = .613)	**.622** ^**a**^	.127 (p = .133)
**9.**	3.79	1.23	**-.185 (p = .028)**	.126 (p = .136)	**.750** ^**a**^	-.138 (p = .103)	.079 (p = .350)	**.507** ^**a**^
**10.**	4.40	0.91	-.120 (p = .161)	-.031 (p = .714)	**.485** ^**a**^	**-.217 (p = .010)**	-.046 (p = .592)	**.259 (p = .002)**
**11.**	2.13	1.26	**.216 (p = .010)**	-.082 (p = .329)	**-.466** ^**a**^	.119 (p = .158)	-.059 (p = .484)	**-.380** ^**a**^
**12.**	2.82	0.81	-.015 (p = .856)	-.024 (p = .781)	**.255 (p = .002)**	-.056 (p = .511)	-.070 (p = .408)	**.192 (p = .023)**
**13.**	1.68	1.02	.086 (p = .312)	.050 (p = .559)	**-.194 (p = .022)**	.067 (p = .436)	-.091 (p = .287)	**-.232 (p = .006)**
**14.**	2.85	1.60	**-.261 (p = .002)**	-.143 (p = .090)	**.316** ^**a**^	**-.251 (p = .003)**	-.079 (p = .350)	**.202 (p = .016)**
**15.**	3.12	1.58	-.018 (p = .831)	-.086 (p = .310)	**.294** ^**a**^	.045 (p = .600)	-.062 (p = .468)	.111 (p = .191)

*Notes*. General opinions

1. I would like to wear a mask in the therapy session.

2. I want the therapist to wear a mask in the therapy session.

3. The mask on my face causes pain and an uncomfortable feeling.

4. The mask makes me feel restricted in my daily life.

5. The mask makes me pay less attention to my appearance in everyday life (e.g., made-up lips and shaving).

6. The mask makes me feel short of breath.

7. Since I can hardly recognize other people’s faces through the mask, I feel insecure in public.

8. Since my face can hardly be seen through the mask, I feel more comfortable in public.

9. The mask protects me from infection.

10. I take the mask requirements in public places very seriously.

11. I think the mask requirement in public places is excessive.

12. I remind other people to wear masks.

13. I always have to be reminded of the mask obligation myself.

14. I mainly use FFP2 masks in everyday life.

15. Since the obligation to wear a mask, I avoid public transport if possible.

^*a*^ *p* < .001.

The point-biserial correlations showed no differences in the subscales as a function of patient gender or different ICD-10 diagnoses. There was a negative correlation between age and the self-confidence expected (pre-version) (r = -.290 p > .001) and experienced (r = .307, p < .001) in therapy by the mask. In addition, there was a positive association between the length of day-hospital or inpatient therapy and self-confidence in therapy expected by the mask (r = .186, p = .027).

As described in the participant section, two cases had significant missing data at the post-measurement time point. To check the robustness of the results, a sensitivity analysis was performed without the cases in question. The conclusions did not change when the cases were excluded from the analysis: The same items were extracted for each factor, and the same significant effects were found (for a summary of the sensitivity analyses, see S4 and S5 Tables).

## Discussion

### Expectations and experiences with wearing face masks

To the best of our knowledge, this is the first study of patients undergoing day hospital or inpatient psychotherapy in which their pre-therapy expectations of wearing a face mask in the psychotherapeutic setting are compared with their final experiences after completion of therapy using a self-developed measurement instrument. Based on the self-developed questionnaire, three factors were extracted regarding the patients’ expectations and experiences: *communication barriers*, *self-confidence*, and *infection control*. Concerning communication barriers, it was found that patients experienced significantly fewer communication barriers in therapy than they first expected. This is consistent with a recent study by Erschens et al. [46], in which wearing a face mask in individual therapy had a relatively small impact on patients’ subjective psychotherapy experiences and the relationship with the psychotherapist from the patient’s perspective. However, these results do not coincide with a more recent study by Grahlow et al. [47], which demonstrated in a large non-clinical sample that emotion recognition is less accurate when wearing a face mask. Reasons why communication barriers, such as not recognizing a smile, feeling distant from the therapist, or poorer acoustic comprehension, were experienced less frequently in our study than feared could be that, as an alternative to facial expressions, other aspects could help recognize emotional expression. For example, in this context, studies found evidence that most basic emotions can also be predicted by ratings of affective vocalization, similar to affective facial signals [48]. In this context, Busso et al. [49], for example, emphasized that emotionality is reflected in both face and speech. Thus, both areas provide perceptual cues to identify positive and negative emotions.

Furthermore, the eyes appear to be an essential feature in face processing. Baron-Cohen et al. [50] developed the well-known “Reading the mind in the eyes” test. They showed that quantifying the ability to decode a person’s mental state based solely on an image of their eyes provides a reliable measure of social cognition. For example, although the most obvious sign of smiling, the lifting of the corners of the mouth, is hidden behind a mask, other signs, namely the lifting of the cheeks and the wrinkles around the eyes that may accompany this movement, remain visible. In this sense, humans can infer various mental states from information in the eye region alone [51,52]. However, as mentioned at the outset, previous literature also provides evidence that women are better at decoding emotions than men, especially from the eye region. The latter should be considered in interpreting the results, given that 72% (N = 102) of the participants in the present study were female. However, particularly regarding the communication barriers expected and experienced by the patients, the time frame in which the data collection took place should also be considered when interpreting the results. It is conceivable that a certain amount of familiarity with wearing face masks could have occurred during the data collection period. For example, the latter could have been achieved by broadening the focus of attention by observers considering the entire body image throughout the pandemic or by focusing more on vocal cues to enable social interactions or differentiation between different emotions in equal measure.

Furthermore, concerning the extracted self-confidence factor, it was shown that some patients expected a higher level of self-confidence in therapy induced by the mask before the start of therapy. They also reported this after the end of therapy.

The latter is consistent with previous literature, which has shown that the mask may appear to have more widespread functionality in certain groups of patients with social difficulties, such as social anxiety. For example, in individuals with social anxiety, the mask has been shown to act as a “safety behavior” by concealing the face (e.g., to cover blushing) and thus reducing fear of negative evaluation [53,54]. Consistent with the latter, a study by Kawagoe and Teramoto [55] in Japan demonstrated a mask-induced reduction of negative emotions in social situations.

In this context, a significant negative correlation was found between age and the expectations and experiences concerning the scale *self-confidence*, i.e., a higher age correlated with a lower relevance of the mask in terms of a self-confidence strengthening function induced by it (among other things, the covering of unloved aspects of one’s face and thus the ability to open up more easily thanks to the mask). A possible explanation for this could be that with increasing age, there is a reduction of self-uncertainty regarding one’s external appearance. Thus, one’s appearance appears to be less relevant in therapy. Furthermore, this also fits with the fact that for older people, mask-induced self-protection against (severe) coronavirus infection has the most significant relevance [56].

There was a positive correlation between the length of hospital stay and expectations of a mask-induced sense of higher self-confidence. One possible interpretation of this correlation could be that the patients who expected to have higher self-confidence in therapy by wearing a mask before starting therapy had more severe structural difficulties at the time of admission, the treatment of which tended to require a more extended time.

### Expectations and experiences of wearing face masks and attitudes in general

In addition, our study shows significant associations between attitudes toward wearing a face mask in general and expectations/experiences of mask-wearing in psychotherapy.

Participants who reported having more negative attitudes toward the mask were more likely to expect and experience negative aspects of wearing a mask in psychotherapy. Participants who felt protected and less restricted by the mask were less likely to expect and experience negative aspects. The latter can be placed in the context of previous studies showing that patients who generally attribute less protection to the mask, experience the COVID-19 pandemic as less risky, and perceive wearing a face mask as a heavy burden also attribute more interactional limitations to wearing a mask.

### Clinical implications and future research

As already described, it has been shown that the extent of the restrictions imposed by wearing a mask in the psychotherapeutic context also appears to depend on the individual patient’s attitude towards wearing a face mask “in general.” Against this background, one clinical implication of the present study could be that, in the context of future therapeutic care, if the wearing of a mask were to be mandatory for patients and therapists, it could make sense to ask patients at the beginning of therapy about their attitude towards wearing a face mask using a short questionnaire to assess better the possible extent of the effects on the therapeutic context and consequently to be able to consider and address them in the context of therapy.

As already described, the communication barrier caused by the mask was experienced by patients in this study as less restrictive than expected before the start of therapy. However, based on the literature, the extent to which wearing a mask makes it more difficult for the other person to perceive emotions remains to be seen. Against this background, to counteract a possible impediment to emotion recognition in the psychotherapeutic context caused by wearing a face mask, one possible approach could be for the therapist to ask more questions about the emotions experienced by patients in the psychotherapeutic setting, thereby encouraging more intensive communication about the emotions experienced in the psychotherapeutic context.

The present study has shown that wearing a mask in the psychotherapeutic context even appears to have had a self-confidence-enhancing function for some patients in the psychotherapeutic context. Against the background of the literature described above, the extent of the latter could also depend on the respective disorder patterns of the patients. Hence, a further evaluation of different disorder patterns is relevant here for further specification of the results. The present study found no correlations between individual ICD diagnoses and the manifestations of the three extracted factors. However, as already described, a patient sample characterized by comorbid disorders was included in the present study in accordance with the treatment setting (see similarities to the study sample of the MEPP study [57]), which consequently needed to allow a precise classification based on individual disorders. Consequently, in this context, the investigation of a sample that is characterized more by individual diagnoses, as is more likely to be found in the outpatient setting, would be recommended in the context of future research to be able to carry out analyses taking into account different patient groups.

In our self-generated questionnaire, we used several items to record the quality of the therapeutic process and the perceived therapeutic relationship from the patient’s perspective. Still, it would have been interesting to analyze the results of the present study. However, it would have been interesting to correlate the results of the present study, i.e., the patients’ scores on the individual factors, with external measuring instruments that record, for example, the quality of the therapeutic process or the quality of the therapeutic relationship, to investigate the extent to which the communication barriers experienced or the increased self-confidence induced by the mask could be dependent on the quality of the therapeutic process or the therapeutic relationship experienced.

## Limitations and conclusion

Our study showed that patients’ experiences with wearing a face mask in psychotherapy were even less negative than expected concerning certain limitations, such as communication barriers. However, patient-specific characteristics also play a crucial role in this context. Our study also has some limitations. First, we used a self-created, non-established questionnaire that was not externally validated. However, the psychometric properties and internal validation were acceptable. A team of therapists also compiled possible restrictions associated with mask-wearing in the therapeutic context from the patient’s perspective and created some items based on this. In this respect, recording possible restrictions directly via patient interviews might have been more advantageous.

Furthermore, all our results were based on patient self-report and did not consider cultural or political aspects that might also have an influence. In addition, we included a mixed patient population. It is possible that the results would have been different with a more homogeneous group of patients selected by diagnoses, for example. The small number of participants must also be taken into account.

Furthermore, research on the expectations and experiences of wearing a face mask from the therapists’ perspective was not considered. In this regard, Erschens et al. [46] concluded in their study, in which some health professionals (not necessarily psychotherapists) also participated, that the face mask tends to evoke negative associations with therapeutic treatment for them. An important alternative to avoiding wearing a face mask in a psychotherapeutic context is telemetric psychotherapy, which became increasingly popular during the pandemic but was also associated with little experience and uncertainty regarding equivalent effectiveness during the pandemic. However, a recent study has shown that the differences between telehealth versus face-to-face interventions in terms of effectiveness were of little clinical significance [58]. In light of these and our results, we conclude that in times of future pandemics, or even in general clinical care, face-to-face therapy should be continued as an alternative to telehealth, taking into account person-specific characteristics, even if wearing a face mask is again required.

## Supporting information

S1 Table Pre- and post-version of the questionnaire. (TIF)

S2 Table Three-factor solution and descriptive statistics for the pre-measurement (22 items). (TIF)

S3 Table Three-factor solution and descriptive statistics for the post-measurement (22 items). (TIF)

S4 Table Three-factor solution and descriptive statistics for the post-version (22 items) excluding the two cases where a large proportion of the values were missing at the post-measurement time point. (TIF)

S5 Table Three-factor solution and descriptive statistics for the post-version (excluding items 11, 12 and 14), excluding the two cases where a large proportion of the values were missing at post-measurement time. (TIF)

S1 Data (SAV)
