# Supplementary figures and images for: Development and testing of a questionnaire on the expectations and experiences with wearing face masks in inpatient and day hospitals
Source: PLoS One. 2024 May 31;19(5):e0304140. doi: 10.1371/journal.pone.0304140 (PMC11142604; doi:10.1371/journal.pone.0304140)

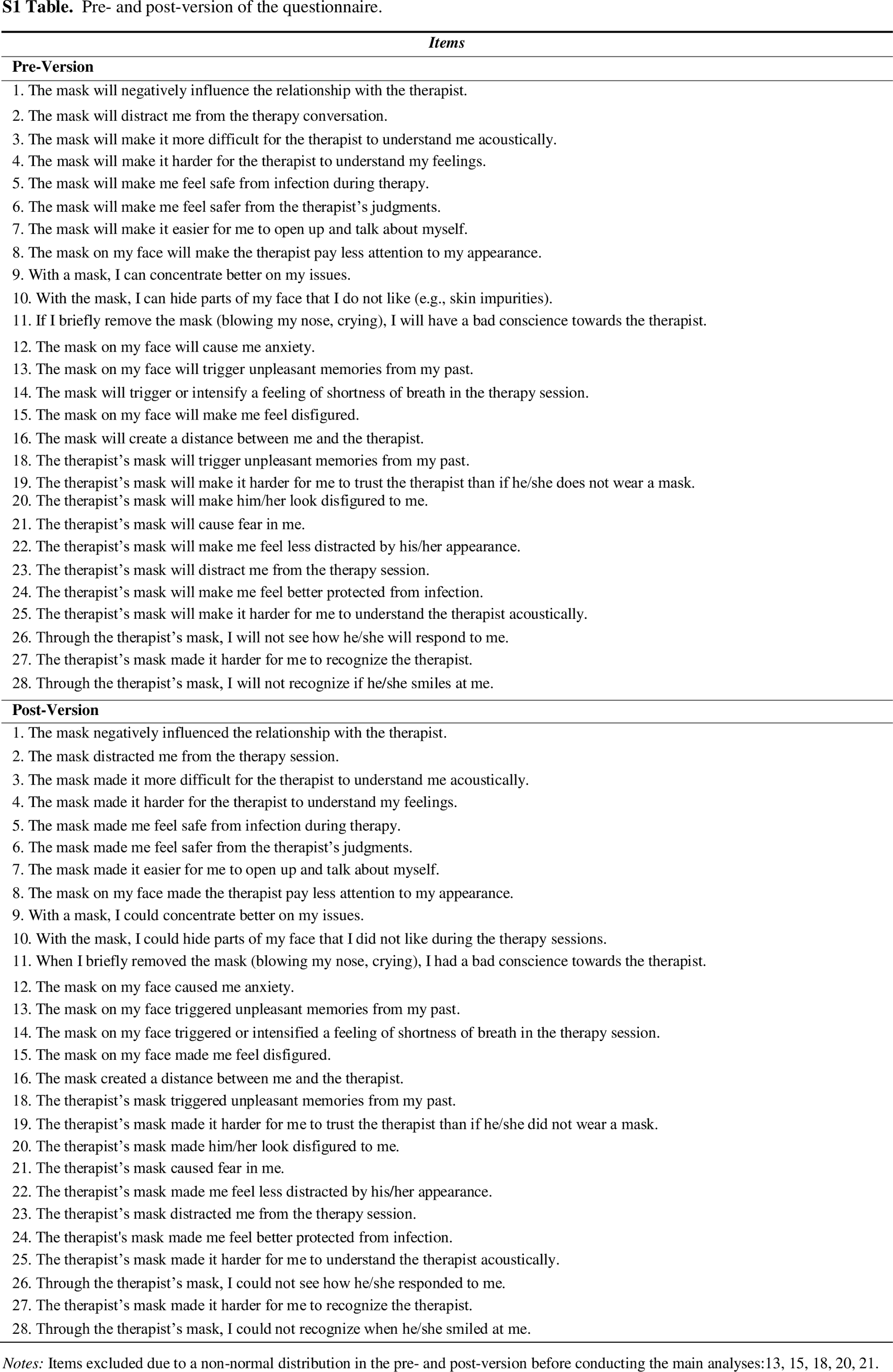

Supplement: S1 Table — (TIF) [file pone.0304140.s001.tif]

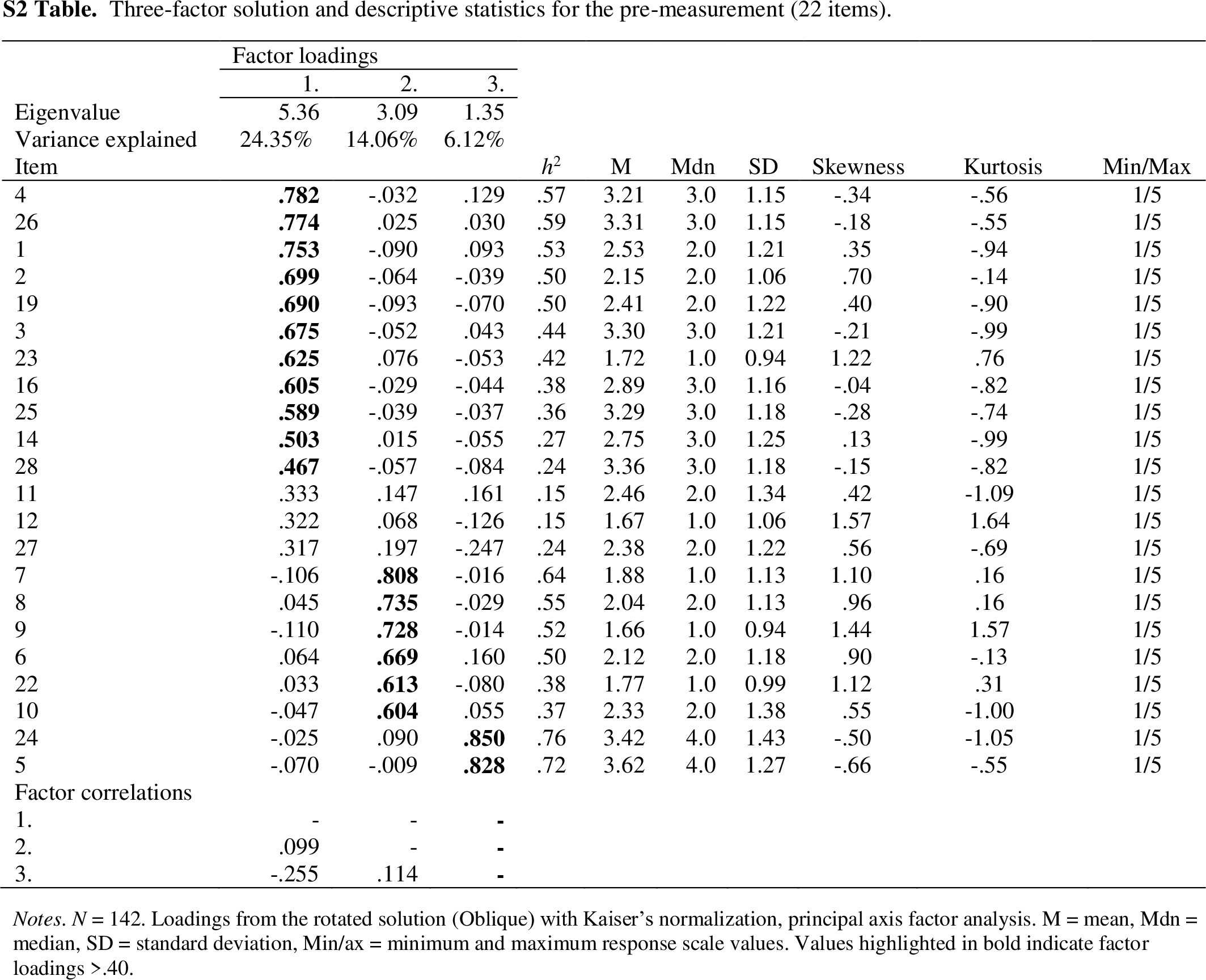

Supplement: S2 Table — (TIF) [file pone.0304140.s002.tif]

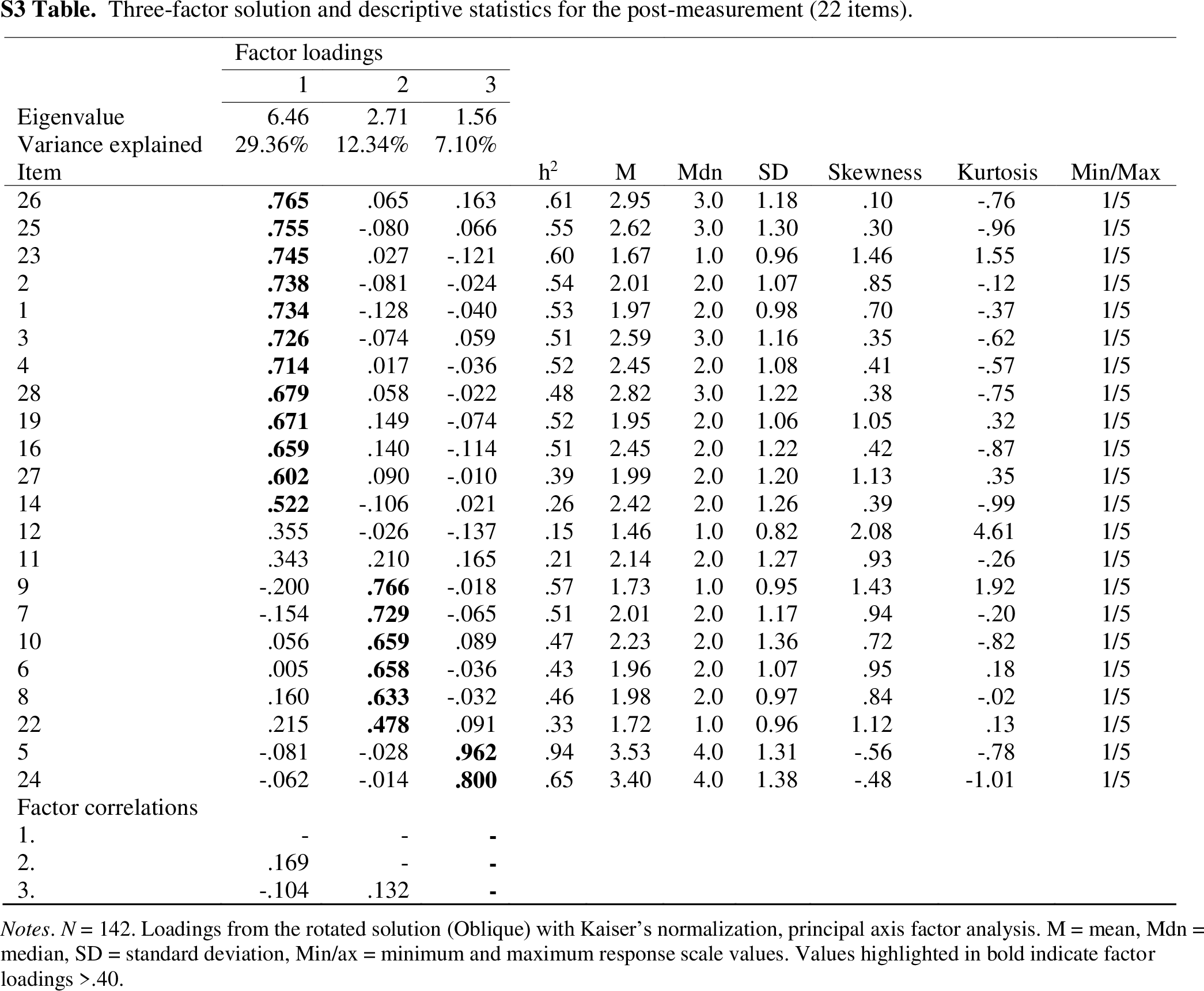

Supplement: S3 Table — (TIF) [file pone.0304140.s003.tif]

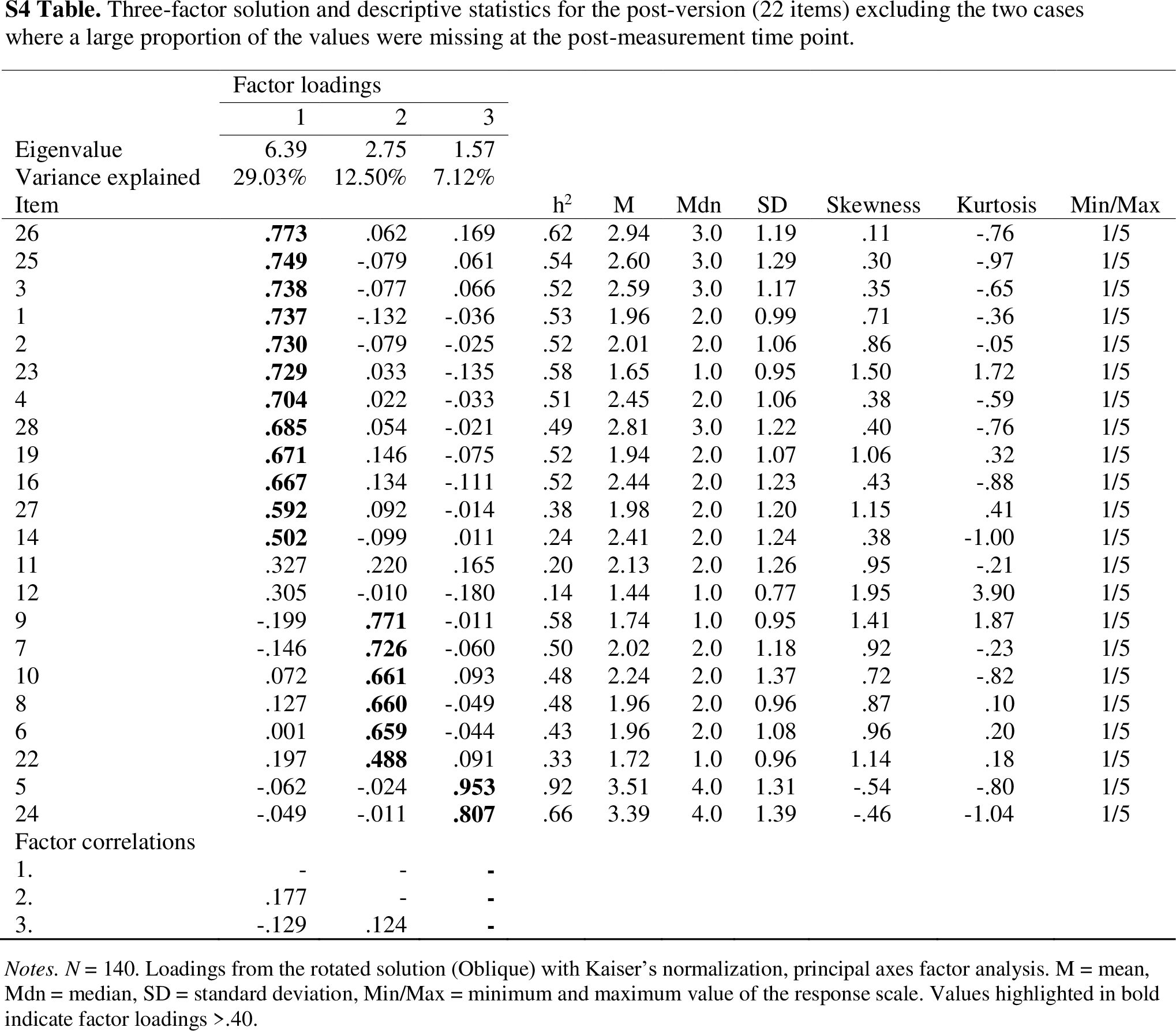

Supplement: S4 Table — (TIF) [file pone.0304140.s004.tif]

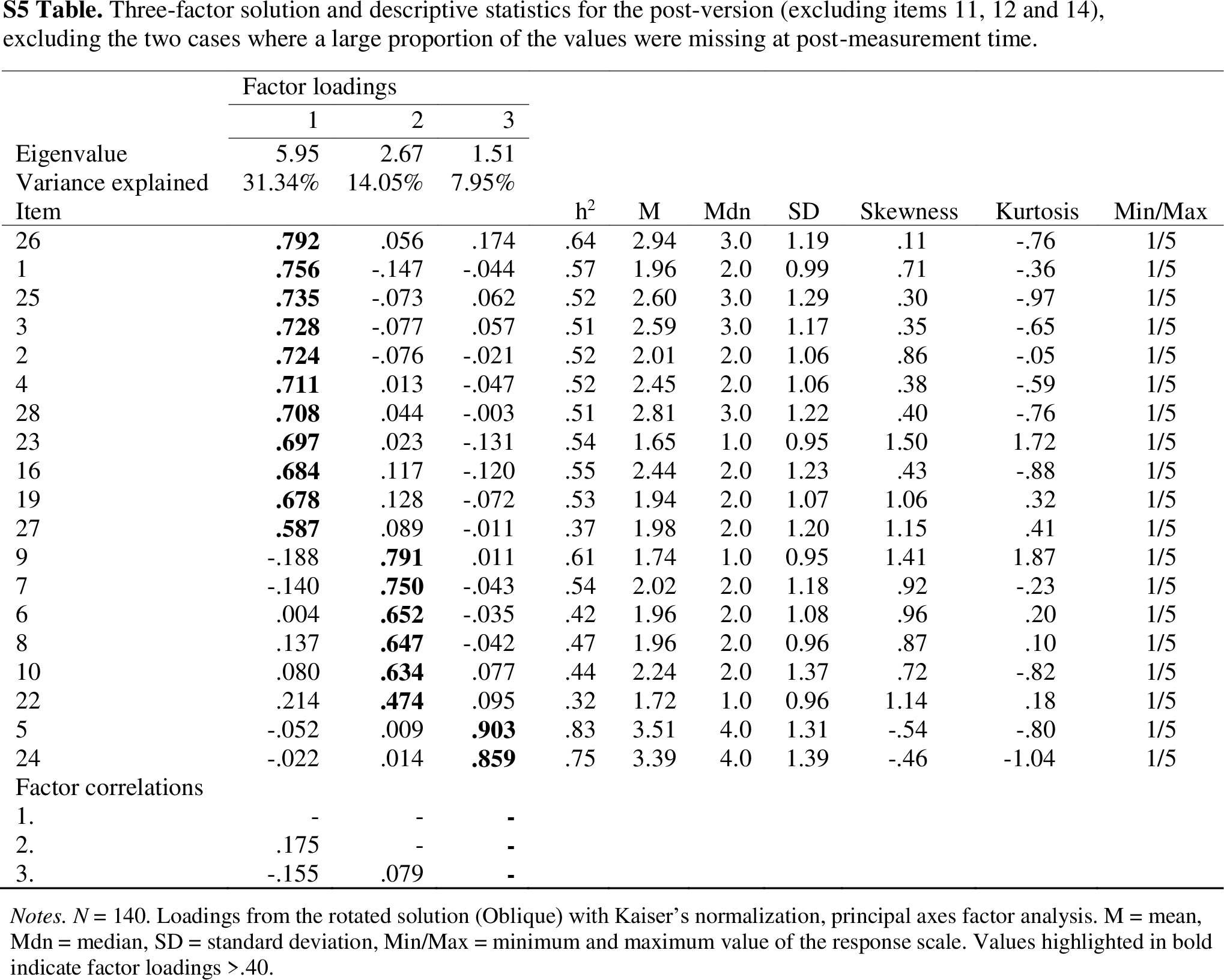

Supplement: S5 Table — (TIF) [file pone.0304140.s005.tif]
